# Muscle Quality Revisited: Biopsy Energetics, MR Spectroscopy, and Muscle Power and Strength

**DOI:** 10.1093/geroni/igab046.482

**Published:** 2021-12-17

**Authors:** Anne Newman, Adam Santanasto, Elsa Strotmeyer, Barbara Nicklas, Paul Coen, Bret Goodpaster, Steve Cummings

**Affiliations:** 1 University of Pittsburgh, Pittsburgh, Pennsylvania, United States; 2 University of Pittsburgh, Pittsburgh, Pittsburgh, Pennsylvania, United States; 3 Wake Forest School of Medicine, Winston-Salem, North Carolina, United States; 4 AdventHealth, Orlando, Florida, United States; 5 California Pacific Medical Center, San Francisco, California, United States

## Abstract

With age, strength may decline faster than muscle mass pointing to a deterioration in muscle quality. Aspects of muscle quality and function are being measured in SOMMA; we hypothesized that in vitro and in vivo bioenergetics capacity of muscle would be related to muscle strength and power. Associations differed between men and women. In men (n=48, ATPMAX, 70, max OXPHOS and max ETS) but not women (n=68, ATPMAX, n=103, max OXPHOS and max ETS), muscle ATP regeneration by 31P MR spectroscopy was correlated with leg power (r = 0.27, p= 0.05). Energy production in tissue was similarly more strongly correlated with power in men than women, though not statistically significant. Correlations between the tissue measures and strength were also stronger in men than women. In ongoing follow-up, we will be able to determine what role that muscle tissue energetics plays in explaining the loss of strength and power with aging.

